# Nail Polish Remover Induced Methemoglobinemia: An Uncommon Occurrence

**DOI:** 10.7759/cureus.32107

**Published:** 2022-12-01

**Authors:** Misbahuddin Khaja, Amber Latif, Irhoboudu Dickson Atogwe, Sachin Bhandari, Pavitra Balasubramanian, Diana M Ronderos

**Affiliations:** 1 Internal Medicine/Pulmonary Critical Care, BronxCare Health System, Bronx, USA; 2 Internal Medicine, University of California, Los Angeles, Los Angeles, USA; 3 Internal Medicine, BronxCare Health System, New York, USA

**Keywords:** cellular hypoxia, environmental toxicology, nail polish cleaner ingestion, dark green urine, g6pd, peripheral cyanosis, methylene blue, methemoglobin

## Abstract

Methemoglobinemia, defined as hemoglobin's impaired oxygen-carrying capacity due to oxidation from the ferrous (Fe2+) state to the ferric (Fe3+) state, has many well-documented etiologies. One example of an uncommon cause of acquired methemoglobinemia is the ingestion of nail polish remover, which can contain methemoglobin generators such as nitroethane, N,N-dimethyl-p-toluidine, and isobutyl nitrite. We present a case of methemoglobinemia in an 81-year-old male following accidental ingestion of isobutyl nitrite-containing nail polish remover, commonly used as a recreational inhalant. Furthermore, we review potentially toxic substances found in commercially available nail products. This case was designed to identify and efficiently treat a rather uncommon cause of methemoglobinemia induced in this case by a common household item, nail polish remover.

## Introduction

Methemoglobinemia (MH) is a potentially life-threatening condition in which hemoglobin’s oxygen-carrying capacity becomes impaired due to oxidation from the ferrous (Fe2+) state to the ferric (Fe3+) state [1]. There are many well-documented congenital and acquired etiologies of MH. While uncommon, cases of MH have been reported from ingestion of nail polish remover and artificial nail remover solutions containing methemoglobin (MetHb) inducers such as nitroethane, N,N-dimethyl-p-toluidine, and isobutyl nitrite [2-4]. Other potentially toxic substances found in these solutions include acetone, toluene, methanol, ethyl acetate, methyl ethyl ketone, and butyrolactone, as well as dyes, oils, and scents that may irritate or corrode the mucosal lining [2].

MH poses many diagnostic hurdles as MetHb interferes with light absorption measured by traditional dual-wavelength pulse oximetry, resulting in inaccurate oxygen saturation. MH’s elusive properties and nonspecific yet potentially fulminant clinical presentation must be well-recognized to provide prompt treatment and avoid long-term health consequences. Methylene blue has been established as the first-line treatment for MH, which, via the nicotinamide adenine dinucleotide phosphate (NADPH)-MetHb reductase pathway, results in conversion to leukomethylene blue, an electron donor that reduces MetHb to hemoglobin [5]. However, methylene blue is not without risk, and precautions must be taken with dosage and comorbidities, most prominently, glucose-6-phosphate-dehydrogenase deficiency (G6PD), to prevent hemolysis and other adverse effects [1].

We present a case of MH, complicated by aspiration pneumonia, induced by the accidental ingestion of a thumb-sized bottle of “Rush” nail polish remover in an 81-year-old male. The patient required a combination of methylene blue, high-flow oxygen, and steroid therapy. This case highlights the need for different approaches, including greater marketing and regulation of products containing the aforementioned substances, enhanced precaution with product use and storage around children and individuals with special care needs, and increased safety precautions in industries where professionals are exposed to these offending agents.

## Case presentation

An 81-year-old male with a past medical history of diabetes mellitus not pharmacologically controlled (hemoglobin A1c 6.3%), peripheral vascular disease, and cerebrovascular accident (four years prior) presented to the emergency department after accidental ingestion of approximately 25 mL of nail polish remover, which he had mistaken for an energy drink, 12 hours before admission. About one hour after ingestion, the patient suddenly experienced severe lightheadedness, which nearly caused him to collapse. The patient decided to come in when the sensation failed to subside by the following morning. The patient denied any associated symptoms, including nausea, vomiting, and blurriness of vision. He also denied depression and mood changes; a review of systems was otherwise negative at the time. The patient admitted to smoking five cigarettes daily for over 40 years and occasionally drinking alcohol. He denied any illicit drug use. He denied surgical history, home medications, and allergies. Family history was non-contributory. He denied following up with a primary care physician.

**Figure 1:**
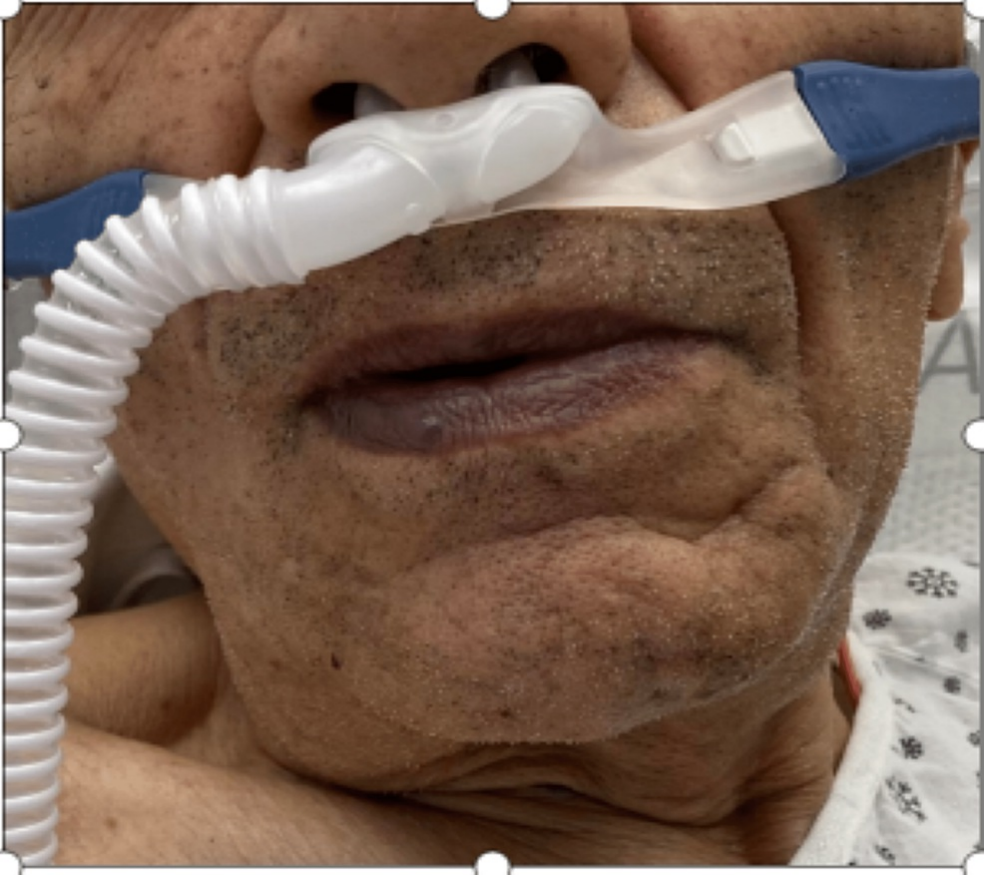
Cyanosis of the lips

**Figure 2:**
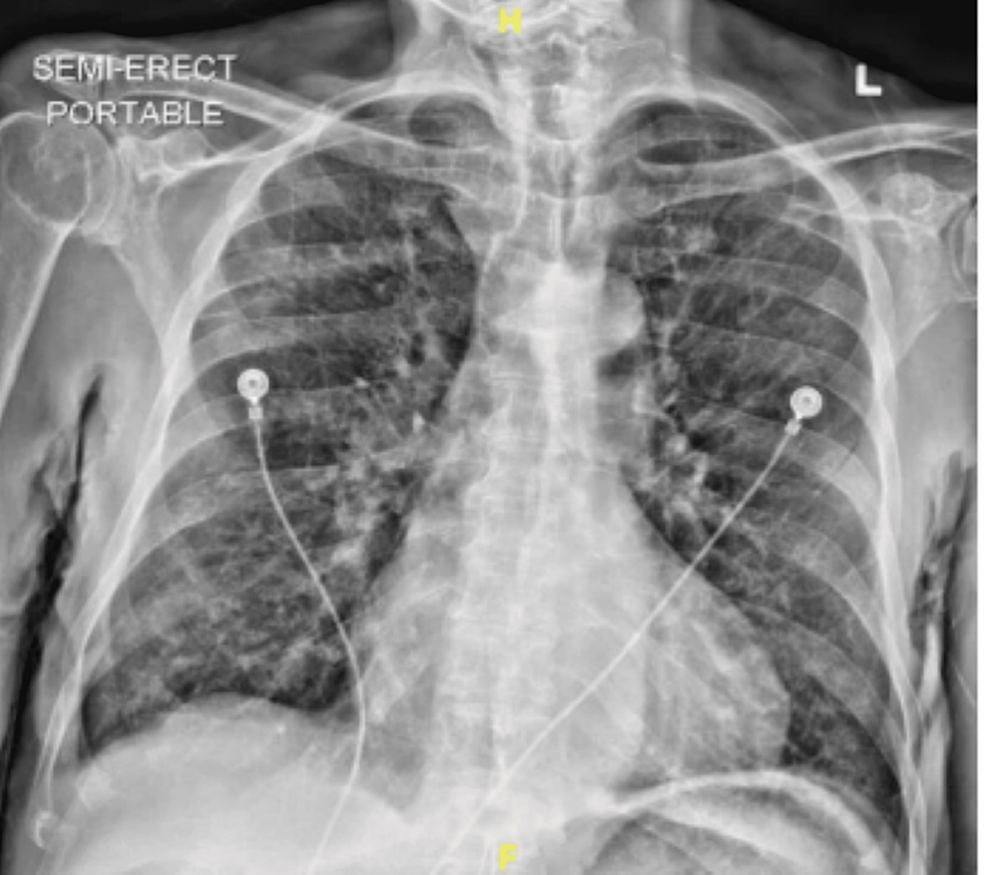
Portable chest x-ray at the time of admission demonstrating no evidence of focal consolidation, pleural effusion, or identifiable acute pulmonary pathology

The patient was tachypneic on initial presentation at 20 breaths/min with oxygen saturation (SpO_2_) of 89% on a non-rebreather mask. Vitals were otherwise stable with a pulse of 88 bpm, blood pressure of 127/70 mmHg, and temperature of 97.7°F. The patient appeared well-developed and in no acute distress. He had blue to black discoloration involving his lips and tongue, with sparing of the gums (Figure 1). He also had blue discoloration of his hands bilaterally, most noticeably at the tips of his fingers. He had bilateral lower extremity edema, hyperpigmentation, and chronic skin changes consistent with his history of peripheral vascular disease. He demonstrated no neurological deficit. His cardiovascular exam was regular in rate and rhythm without murmurs, rubs, or gallops. His abdomen was soft, non-tender, and non-distended. Chest x-ray on initial presentation demonstrated no focal consolidation, pleural effusion, or pneumothorax (Figure 2). An electrocardiogram revealed sinus bradycardia (54 bpm) and a right bundle branch block with no previous ECG for comparison.

Poison control was contacted immediately upon patient stabilization with oxygen supplementation. The team was informed that nail polish remover is primarily acetone based, and severe cases can manifest with CNS depression, hypotension, metabolic acidosis, and seizures. Therefore, poison control recommended obtaining acetone levels, serum electrolytes, and blood gas levels while managing the patient symptomatically. The patient's laboratory values on admission are presented in Table 1.

**Table 1:** Patients' lab values on admission BUN: blood urea nitrogen, AGAP: anion gap, GFR: glomerular filtration rate, PCO_2_: partial pressure of carbon dioxide, PO_2_: partial pressure of oxygen, U/G: enzyme unit per gram.

Parameter	Patients' values	Normal range
WBC	10.3 k/uL	4.8-10.8 k/uL
Neutrophil %	79%	40%-70%
Hemoglobin	14 g/dL	12.0-16.0 g/dL
Hematocrit	41.80%	42.0%-51.0%
Platelet	229 k/uL	150-400 k/uL
Sodium	137 mEq/L	135-145 mEq/L
Potassium	4.7 mEq/L	3.5-5.0 mEq/L
BUN	23.0 mg/dL	8.0-26.0 mg/dL
Creatinine	1.0 mg/dL	0.5-1.5 mg/dL
AGAP	12 mmoles/L	9-15 mmoles/L
GFR	56 ml/min/1.73 m^2^	35-81 ml/min/1.73 m^2^
Venous pH	7.385	7.350-7.450
PCO_2_	52.2 mmHg	35.0-45.0 mmHg
PO_2_	17.2 mmHg	83.0-108.0 mmHg
Bicarbonate	30.5 mmoles/L	22.0-28.0 mmoles/L
Arterial pH	7.39	7.350-7.450
PCO_2_	43.4 mmHg	35.0-45.0 mmHg
PO_2_	297.0 mmHg	83.0-108.0 mmHg
O_2_ saturation	98%	95%-98%
Bicarbonate	25.7 mmoles/L	22.0-28.0 mmoles/L
Lactic acid	1.5 mmoles/L	0.5-1.6 mmoles/L
Methemoglobin	39.90%	0%-2.0%
Glucose-6-phosphate dehydrogenase	17.7 U/G Hgb	7.0-20.5 U/G Hgb
Serum acetone	Negative	Negative
Acetaminophen	Negative	Negative
Acetylsalicylic acid	Negative	Negative
Ethanol	Negative	Negative
Carboxyhemoglobin	Negative	Negative
Urinalysis	Unremarkable	Unremarkable
Urine toxicology	Negative	Negative

**Figure 3:**
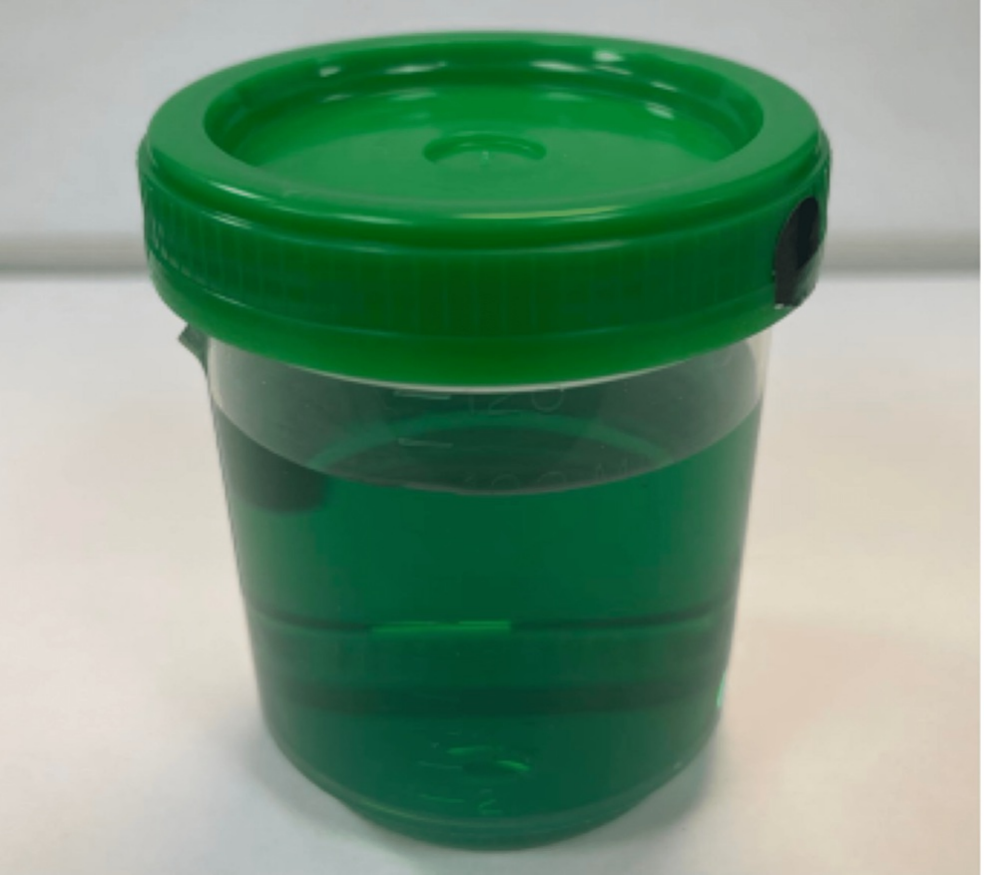
Dark green-tinted urine of a patient receiving methylene blue therapy after accidental ingestion of nail polish remover

The patient was diagnosed with methemoglobinemia (MH). Upon follow-up with poison control, it was recommended to administer methylene blue at a rate of 1 mg/kg over five to 30 minutes before rechecking the MetHb level to assess the necessity of a repeat dose. The patient was admitted to the ICU to manage acute hypoxemic respiratory failure, where he was given a one-time 75 mg IV push of methylene blue. Within two hours of administration, his MetHb level was reduced from 39.9% to 3.9%. The patient experienced two episodes of coffee-ground emesis during treatment. He was started on a proton pump inhibitor (PPI), IV azithromycin, and ceftriaxone due to suspicion of aspiration pneumonia. The patient demonstrated marked improvement in respiratory status overnight and resolution of cyanotic discoloration. His urine was noted to have dark green pigmentation during this period, commonly associated with methylene blue therapy (Figure 3). The patient was transitioned to nasal cannula and moved out of the ICU to the medical ward. Shortly after that, the patient insisted on discharge for varying personal reasons. The patient was evaluated by psychiatry, who diagnosed him with adjustment reaction, and deemed his decisional capacity intact. The patient was ultimately discharged against medical advice approximately 36 hours after admission. He was given a prescription for azithromycin and cefpodoxime and instructions to follow up in the clinic and establish care with a primary care physician.

## Discussion

MH is a potentially life-threatening condition in which hemoglobin's oxygen-carrying capacity becomes impaired due to oxidation from the ferrous (Fe2+) state to the ferric (Fe3+) state. MetHb physiologically forms in the body at a rate of 3% per day and is maintained under 1% by multiple protective mechanisms [1]. Congenital forms of MH can manifest due to autosomal recessive defects in cytochrome b5 reductase, the pathway responsible for 95%-99% of endogenously produced MetHb, or autosomal dominant mutations in genes coding for globin proteins, hemoglobins M [1]. There are numerous well-documented acquired MH etiologies, with dapsone and benzocaine being the most commonly used agents. Other acquired causes include exposure to herbicides, pesticides, exhaust fumes, industrial chemicals, and many pharmaceuticals (chloroquine, metoclopramide, nitroglycerin, and sulfonamides to name a few) [3]. One less common cause of acquired MH is ingesting certain nail polish remover and artificial nail remover solvents. Therefore, it is essential to understand the potential toxicities regarding the ingestion of substances used in these solvents to identify and promptly treat MH and avoid a potentially lethal course.

Nail polish remover and artificial nail remover products marketed in the USA generally contain ingredients including acetone, isobutyl nitrite, nitroethane, N,N-dimethyl-p-toluidine, toluene, methanol, ethyl acetate, methyl ethyl ketone, and γ‐butyrolactone. In addition, these solutions often contain other additives such as colorings, soothing oils, and scents that may also be irritating or corrosive to the mucosal lining. These products can be separated into two categories, including acetone-based and acetone-free alternatives.

The active ingredient acetone, a colorless chemical with a distinct odor, is naturally found in the environment and commonly utilized industrially and in household products. Acetone toxicity is rare; exposure usually occurs via ingestion, inhalation, or direct skin absorption. It is primarily metabolized in the liver and excreted through the lungs. In the 2019 Annual Report of the American Association of Poison Control Centers National Data Collection System, only one in 1,553 cases of acetone exposure was reported to have resulted in death [4]. In very small quantities, acetone exposure is unlikely to have serious health consequences. However, larger quantities and chronic exposure have reportedly resulted in bronchial edema, respiratory distress, cardiovascular collapse, acute kidney injury, anion gap metabolic acidosis, leukocytosis, central nervous system depression, coma, and seizure [5]. Due to the potential toxicities associated with acetone ingestion, coupled with acetone’s effects on the hands, including nail brittleness and contact dermatitis, acetone-free alternative nail products have risen in popularity in recent years.

While it is widely perceived that acetone-free nail polish removers and artificial nail removers are safer for public use, these alternative solutions can contain ingredients such as isobutyl nitrite, nitroethane, and N,N-dimethyl-p-toluidine which carry an arguably more sinister set of risks. It was determined upon further investigation that this patient’s symptoms were caused by ingestion of “Rush” nail polish remover, a product containing the ingredient isobutyl nitrite that is marketed as a nail polish remover or cleaning product but is instead used recreationally and for sexual enhancement. These energy drink-like glass vials, labeled “Rush”, “Bolt”, “Jungle Juice”, and casually referred to as “poppers”, are often found in adult stores. Inhalation of this recreational substance induces vasodilation and a state of euphoria followed by reflex tachycardia, which, in combination, can create feelings of enhanced sexual pleasure. When ingested, isobutyl nitrite, with its substantial oxidizing properties, induces a more fulminant course of symptoms and can progress to MH [3]. Nitroethane is occasionally used as a solvent in nail polish removers and is more commonly used in artificial nail removers. Its colorless appearance and fruity odor often cause it to be mistaken for acetone. Nitroethane is a weak inducer of MetHb as it gets metabolized to the strong oxidizer nitrite (NO_2_), and then eventually to nitrate (NO_3_). Nitroethane ingestion can result in the prolonged formation of MetHb, requiring additional dosing of methylene blue. Therefore, patients admitted for nitroethane toxicity should have an additional period of observation post-treatment. [4]. Ingestion and inhalation of N,N-dimethyl-p-toluidine, a commercially available product used in artificial nail solutions and dental and bone cement hardeners, have been linked to MH. It is biochemically oxidized to the metabolite phenylhydroxylamine, a potent and well-documented producer of MetHb [6]. A case of MH has also been reported in a two-year-old child who ingested nail polish remover containing methanol, ethanol, and ethyl acetate, ingredients which are not independently believed to induce MH directly but may potentially, in combination, produce a toxic metabolite [7].

Although other substances commonly found in these solvents have not been directly proven to induce MetHb, it should be noted that they are not without risk. Ethyl acetate, for example, has been demonstrated to cause CNS depression and liver congestion in high concentrations, respiratory distress, anemia, and leukocytosis with chronic exposure. One notable incident in a group of runners displaying shortness of breath, wheezing, and coughing was traced back to ethyl acetate and toluene usage in their practice facility, two ingredients commonly found in nail polish remover solvents. The ethyl acetate and toluene concentrations were below the threshold for federal guidelines but sufficient to induce respiratory distress. Methyl ethyl ketone is an oxidizing agent used in many nail solvents, and its ingestion has been linked to corrosive burns leading to inhalational pneumonitis and acidosis. Another case of severe toxicity was reported in a nine-month-old boy sucking on acetone-free nail polish remover pads for less than one minute. The γ‐butyrolactone contained in the product was converted into γ‐hydroxybutyrate, a well-documented central nervous system depressant that caused hypotension, bradycardia, and respiratory acidosis [3]. There is no scientific basis to demonstrate that acetone-free nail polish remover alternatives are safer for public consumption than acetone-based solutions.

It is important to be prepared to differentiate between the various presentations resulting from the ingestion of these chemicals, as MetHb inducers typically precipitate a nonspecific and more insidious course compared to the more directly corrosive agents discussed previously. Patients with MH often present with sudden-onset cyanosis and hypoxia on pulse oximetry that does not improve with supplemental oxygen administration, and metabolic acidosis is a likely outcome. Symptom intensity is primarily dependent on the level of MetHb [3]. Cyanosis is clinically evident in most people when MetHb reaches 10%, and further increases lead to tissue hypoxia. The classic “chocolate brown” appearance of blood with a high concentration of MetHb can be noted at around 15%. Anxiety, light-headedness, and cephalgia present as low as 20%, and tachypnea, loss of consciousness, and altered mental status present around 30%. A MetHb level of 50% poses a risk of dysrhythmia, seizures, or a comatose state. Fatality can occur when MetHb reaches 70% [8].

MH poses a diagnostic challenge as traditional dual-wavelength pulse oximetry devices demonstrate inaccurate readings in the setting of MH, and calculated oxygen saturation of arterial blood (SaO_2_) is falsely normal. Pulse oximetry studies light absorbance at 660 and 940 nm wavelengths to measure the ratio of oxyhemoglobin to deoxyhemoglobin. MetHb causes interference due to its high absorbance at both wavelengths, resulting in a distortion in this ratio. Once MetHb reaches a level of 30%, the absorbance ratio becomes one, resulting in SpO_2_ trending toward a plateau of 85% [1]. An arterial blood gas study may reveal the characteristic chocolate-brown color of blood resulting from MetHb content. Oxygen saturation calculated from an arterial blood gas is derived from the partial pressure of oxygen dissolved in the blood, a value that is affected by many variables but is independent of MetHb content. This calculated oxygen saturation often contrasts the value obtained by pulse oximetry, lending to a saturation gap above 5% consistent with MH. Lack of peripheral oxygen saturation improvement with supplemental oxygen administration is another diagnostic clue. MH can be confirmed by a direct MetHb blood study or by the Evelyn-Malloy test in which cyanide is bound to the positively charged MetHb, thereby eliminating absorption at the 630-635 nm wavelength proportional to MetHb, allowing for quantification of the percentage of total hemoglobin concentration [9]. Cooximetry utilizes multiple light wavelengths to distinguish hemoglobin states and may be a valuable tool in observation, though it is limited by cost and availability.

The first step in managing MH is the causative agent's withdrawal. Mild cases of MH resolve with endogenous reduction and supportive therapy such as intravenous fluid administration. Methylene blue is the first-line treatment of MH, indicated when MetHb exceeds 20% or if the patient is symptomatic, and it acts by accelerating MetHb reduction via the NADPH-MetHb reductase pathway. Methylene blue is reduced to leukomethylene blue, which then acts as an electron donor, converting MetHb to hemoglobin. It is administered intravenously at a rate of 1-2 mg/kg over five minutes, with the maximum effect occurring at 30 minutes [10]. An additional dose may be warranted after one hour if symptoms or MetHb levels remain above the threshold for intervention. Risks of methylene blue therapy include hemolysis and a paradoxical increase in MetHb in high doses exceeding 7 mg/kg. Known or suspected glucose-6-phosphate-dehydrogenase deficiency (G6PD), an X-linked recessive hereditary disease, is a contraindication to methylene blue therapy as it serves as the key enzyme in the NADPH-generating pathway, resulting in insufficient NADPH levels for methylene blue reduction [11]. Therefore, screening for G6PD should take place before therapy initiation. In confirmed cases of G6PD, exchange transfusions, ascorbic acid therapy, and hyperbaric oxygen administration have been demonstrated as effective alternative management modalities [12].

## Conclusions

Patients presenting with acquired MH may present with widespread, nonspecific symptoms. This case and other similar cases of MH resulting from the ingestion of nail polish remover and artificial nail solvents demonstrate a need for heightened suspicion for providers when managing patients presenting with cyanosis and hypoxia. While the true incidence of acquired MH is unknown, a good history is essential to suspect the diagnoses in patients, particularly in those who may not realize the potential toxicity associated with everyday products. 

This case also suggests that several actions be taken to reduce ingestion, and product manufacturers should improve marketing strategies to better inform the general public on the harm associated with the ingestion of products that contain these harmful substances, especially since they are within reach of the general society. Conclusively, prompt recognition and early intervention of MH are crucial to prevent long-term health complications and a potentially lethal course.
